# Editorial: Tumor Heterogeneity

**DOI:** 10.3389/fmed.2019.00156

**Published:** 2019-07-05

**Authors:** Stefano La Rosa, Laura Rubbia-Brandt, Jean-Yves Scoazec, Achim Weber

**Affiliations:** ^1^Institute of Pathology, University Hospital and University of Lausanne, Lausanne, Switzerland; ^2^Division of Clinical Pathology, University Hospitals, Geneva, Switzerland; ^3^Department of Pathology, Gustave Roussy Cancer Campus, Villejuif, France; ^4^Faculté de Médecine de Bicêtre, Université Paris Sud, Le Kremlin-Bicêtre, France; ^5^Department of Pathology and Molecular Pathology, University and University Hospital Zurich, Zurich, Switzerland; ^6^Institute of Molecular Cancer Research, University of Zurich, Zurich, Switzerland

**Keywords:** tumor heterogeneity, intra-tumor heterogeneity, inter-tumor heterogeneity, pathology detection, carcinoma, follicular lymphoma, adrenocortical tumor

Cancer should not be considered just as an agglomerate of cells, but rather as a complex morpho-functional entity with different compartments, which represent all together the so-called intratumor heterogeneity (ITH), often difficult to identify using a morphological approach alone. In the era of personalized medicine, the identification of ITH is an emerging issue for pathologists because it is strongly related to clinical oncology influencing clinical presentation, symptoms, behavior, tumor classification, and response to therapy (1, 2). ITH is a spatial and temporal phenomenon generally distinct into two parts: clonal heterogeneity, which is due to the different distribution of molecular alterations, and non-clonal heterogeneity that depends on the interaction between tumor and surrounding microenvironment. Stanta and Bonin have clearly summarized the different levels of ITH, which include a wide spectrum of molecular alterations (gene mutations, promoter genes methylation, copy number alterations) that directly influence morphological differentiation, immunophenotype and, in turn, clinical presentation, biological aggressiveness, and response to therapy.

In addition to ITH, the concept of tumor heterogeneity (TH) also includes intertumor heterogeneity. It has been referred either to the differences observed between a primary cancer and related metastases in the same patient or to the differences observed in different patients bearing tumors with the same morphological features and consequently diagnosed under the same specific entity (also defined interpatient heterogeneity) (3, 4).

The diagnostic impact of both intra- and inter-tumor heterogeneity must be taken into account by both pathologists and clinicians. Indeed, the representativeness of small biopsy samples and fine needle aspirates might be challenged by tumors with a strong degree of ITH. Results obtained by the examination of only one tumor site in disseminated metastatic cancer disease might be not valid in another tumor site from the same patient, i.e., between primary and metastases, or between metastases. This is especially the case for metastases presenting long after the diagnosis of the primary, hence the current debate about the importance, benefits and indications of “re-biopsies” during the course of the disease.

Breast cancer is an excellent example of both intra and intertumor heterogeneity that strongly influence the choice of the more appropriate clinical management. This mainly depends on the evaluation of several different parameters, namely hormone receptors expression and HER2 amplification, which can differ from patient to patients but also in areas of the same tumor (spatial heterogeneity) also during its progression over time (temporal heterogeneity) (Turashvili and Brogi).

“Colorectal cancer is not just colorectal cancer” is a tremendously effective paraphrase proposed by Blank et al. to underlying that tumor heterogeneity, also in this context, directly impacts the therapeutic strategy and patients' outcome. Colorectal carcinoma is an excellent example recapitulating the large spectrum of clonal and, especially, non-clonal heterogeneity. Non-clonal heterogeneity represented by tumor interaction with the surrounding microenvironment reflects some morphological features easily recognizable in routine H&E preparations (i.e., vascularization, infiltrating-tumor associated inflammatory cells, and tumor deposits in surrounding fat tissue) and plays a pivotal role in tumor development directly influencing gene expression in cancer cells.

TH is not a peculiar feature of solid tumors, but it plays a pivotal role in the biology of lymphomas as well, representing a key aspect that influences the clinical management at different time points during the course of the disease. Magnoli et al. have reviewed the molecular cytogenetic and immunohistochemical heterogeneity of follicular lymphoma, a paradigmatic disease traditionally considered as a single monolithic entity. Clusters of gene mutations and deregulated epigenetic mechanisms (clonal ITH), in concert with the interaction between neoplastic lymphoid cells and microenvironment (non-clonal ITH), determine the outcome and, consequently, greatly influence the therapeutic choices. Data reported and critically reviewed strongly suggest that follicular lymphoma is a plastic disease from its early steps of development to progression and final transformation.

TH is mainly referred to malignant neoplasms where it has been largely investigated in the last years. However, it is worth noting that TH is a phenomenon that may also play a crucial role in benign neoplasms as described by Mete and Duan in benign adrenocortical tumors. Specific genetic alterations determine different histopathological features and greatly influence different presentations of both primary hyperaldosteronism and Cushing disease. This review clearly summarizes the genotype-phenotype correlations elucidating the biological background of complex endocrine diseases that can clinically present as different faces of the same entity.

Once it has been established that tumor heterogeneity at its different levels (molecular, immunophenotypic, morphological, and clinical) represents a crucial point in the understanding of tumor biology, the last but not least challenge for pathologists is the methodological approach to its identification and evaluation in daily routine practice. Tumor sampling methods follow standardized and accepted protocols, which vary depending on the specific topography (5). These protocols have been created with the assumption that samples selected at gross examination must be representative of the whole neoplasm. Although this approach is true for diagnostic purposes, it clearly seems to be inadequate to identify ITH. At this regard, Stanta and Bonin describe a sampling procedure, which includes different samples in the border, sub-border and central part of the tumor with the aim to cover different areas as much as possible. A similar approach is well-described by Cortés et al. who describe in detail a multisite tumor sampling approach that seems a promising technique useful to detect ITH, not adding extra-costs and extra-time. Finally, it is worth noting that liquid biopsy approaches are currently evaluated as promising tools to overcome the limitations inherent to spatial and temporal TH (6).

## Author Contributions

All authors listed have made a substantial, direct and intellectual contribution to the work, and approved it for publication.

### Conflict of Interest Statement

The authors declare that the research was conducted in the absence of any commercial or financial relationships that could be construed as a potential conflict of interest.
